# Resveratrol Modulates the Topoisomerase Inhibitory Potential of Doxorubicin in Human Colon Carcinoma Cells 

**DOI:** 10.3390/molecules191220054

**Published:** 2014-12-01

**Authors:** Anika Schroeter, Doris Marko

**Affiliations:** Department of Food Chemistry and Toxicology, University of Vienna, Währinger Str. 38, 1090 Wien, Austria; E-Mail: anika.schroeter@univie.ac.at

**Keywords:** resveratrol, doxorubicin, colon cancer, topoisomerase

## Abstract

Resveratrol (RSV) is currently being widely discussed as potentially useful for anticancer therapy in combination with classical chemotherapeutics, e.g., the topoisomerase II (TOP II) poison doxorubicin (DOX). However, there is still a lack of knowledge of possible interference at the target enzyme, especially since RSV itself has recently been described to act as a TOP poison. We therefore sought to address the question whether RSV affects DOX-induced genotoxic and cytotoxic effects with special emphasis on TOP II in HT-29 colon carcinoma cells. RSV was found to counteract DOX-induced formation of DNA-TOP-intermediates at ≥100 µM for TOP IIα and at 250 µM for TOP IIβ. As a consequence, RSV modulated the DNA-strand breaking potential of DOX by mediating protective effects with an apparent maximum at 100 µM. At higher concentration ranges (≥200 µM) RSV diminished the intracellular concentrations of DOX. Nevertheless, the presence of RSV slightly enhanced the cytotoxic effects of DOX after 1.5 h and 24 h of incubation. Taken together, at least in cell culture RSV was found to affect the TOP-poisoning potential of DOX and to modulate its cytotoxic effectiveness. Thus, further studies are needed to clarify the impact of RSV on the therapeutic effectiveness of DOX under *in vivo* conditions.

## 1. Introduction

Resveratrol (RSV) is a naturally occurring polyphenol, which can be found in a variety of plants such as grapes, peanuts and berries. It has been shown that RSV possesses antiproliferative effects in a broad range of different cancer cells by influencing (amongst others) pathways involved in cell cycle progression and apoptosis induction [1,2,3]. Recently, Leone *et al.* [4] reported that RSV exhibited poisoning potential against topoisomerase II (TOP II) in human glioma cells. TOP are highly conserved enzymes that are essential for the maintenance of DNA integrity during all processes affecting DNA topology such as replication, transcription or repair. Two isoforms of TOP II are expressed in humans: TOP IIα and TOP IIβ. Both are capable of removing knots and preventing over- and underwinding of the double helix by generating transient double strand breaks. To assure genomic stability during this intermediate DNA cleavage step the enzyme is covalently linked to its substrate DNA, a state, which is called “cleavage complex” [5,6]. TOP-targeting compounds can affect the catalytic cycle in different stages. So called TOP poisons stabilize the cleavable complex, thus trapping the enzyme covalently linked to the DNA. As a consequence severe DNA damage occurs, which is hence used by a range of clinically used chemotherapeutics like e.g., doxorubicin (DOX) [7]. Altogether the chemopreventive and anticarcinogenic potential of RSV makes it a promising candidate for clinical trials as a single compound but also in combination with commercial chemotherapeutics like DOX. 

The dosage of DOX during chemotherapy is limited by side effects as cardiotoxicity, as well as by DOX-resistant cancer types. In recent years many attempts have been made to overcome the resistance, for example by a combination of the chemotherapeutic with plant polyphenols [8]. Several studies investigated a combination of DOX and RSV with promising results. On the one hand RSV seems to have protective effects against DOX-induced cardiotoxicity and on the other hand RSV might help to sensitize cancer cells against DOX-induced toxicity [9,10,11,12,13,14]. A combination of the two substances during chemotherapy is therefore discussed as a promising future approach. On the other hand, during the last decade reports on potential beneficial effects of RSV with respect to chemoprevention strongly promoted the market of respective supplements [15]. Clinically uncontrolled consumption of RSV supplements during DOX-based chemotherapy is therefore not to be dismissed. Despite of the growing interest of potential interaction of these two substances, to our knowledge, no study has looked into the combinatory effects of DOX and RSV on TOP II, so far. Therefore, we addressed the question whether RSV, as a newly described TOP poison, affects the TOP-targeting potential of DOX with special emphasis on the consequences for DOX-induced genotoxicity, intracellular DOX concentration and cytotoxicity. Considering the expected low systemic bioavailability of RSV, the well characterized human colon cancer cell line HT-29, originating from the intestinal tract, was selected as a model system.

## 2. Results and Discussion

### 2.1. Topoisomerase Inhibition in HT-29 Cells

**Figure 1:**
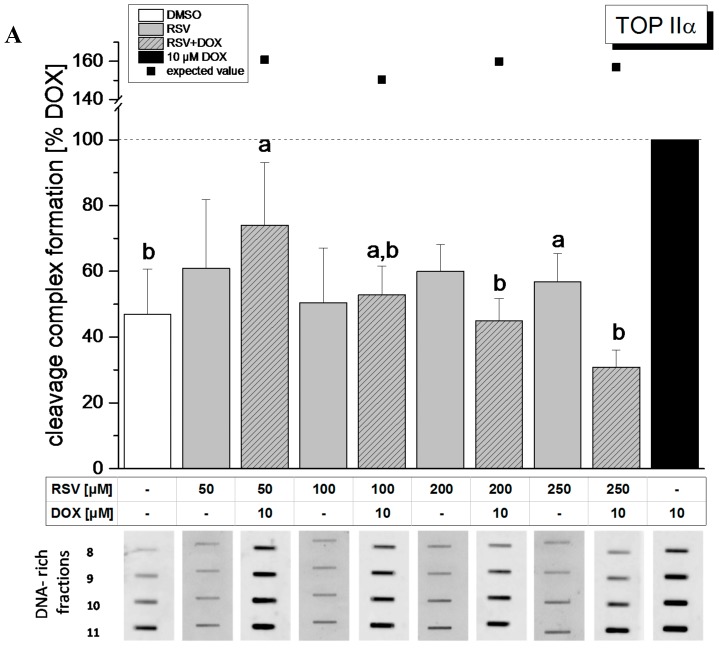

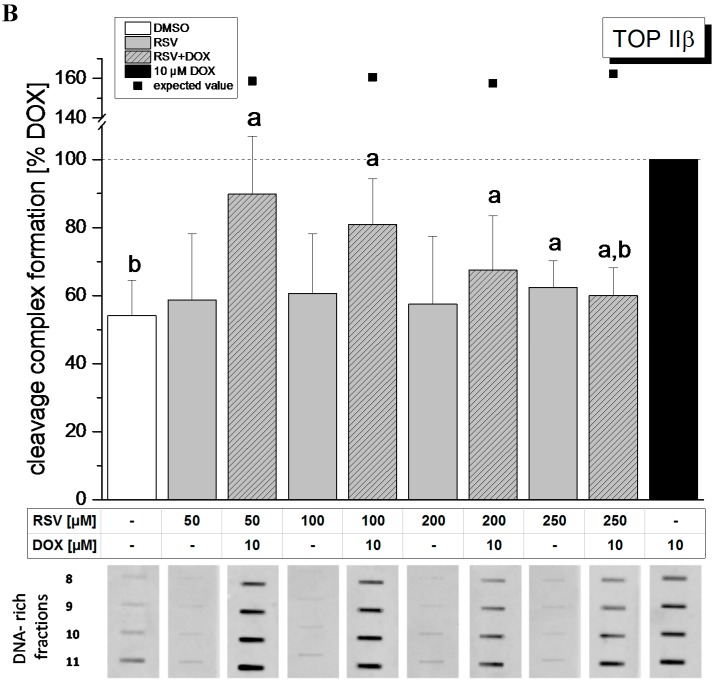
“Isolating *in vivo* complexes of enzyme to DNA” (ICE assay) for assessment of the level of topoisomerase (TOP) II covalently linked to DNA after co-incubation of HT-29 cells with resveratrol (RSV) and doxorubicin (DOX). Cells were pretreated with RSV in the indicated concentrations for 30 min, followed by 60 min of co-incubation together with 10 µM of DOX or incubated with the single compounds. Displayed are representative blots out of six independent experiments, which depict the cleavage complexes formed after incubation with the test substances or DMSO (solvent control) in the DNA-rich fractions for TOP IIα (**A**) and TOP IIβ (**B**). The chemiluminescence signal was quantified in relation to DOX and statistically analyzed by One-way ANOVA followed by Fisher’s least significant difference (LSD) test. Significances indicated as “a” refer to a comparison to DMSO, significances indicated as “b” to a comparison to DOX. All significances refer to a statistical level of *p* < 0.05.

DOX is a well described TOP II poison. The members of this class of TOP-targeting compounds act by increasing the concentration of cleavage complexes in the cells. To investigate the combinatory effects of RSV and DOX on the TOP II-DNA cleavage complex formation the “isolating *in vivo* complexes of enzyme to DNA” assay (ICE assay) was performed in HT-29 cells (Figure 1). 

Incubation with DOX significantly increased the proportion of cleavage complexes in comparison to the solvent control DMSO (Figure 1A,B), which is in line with previous publications of the DOX poisoning potential in HT-29 cells [16,17]. Treatment with rising concentrations of RSV as a single compound resulted in a weak increase of TOP II complex formation, which was significant only in the highest tested concentration of 250 µM for both isoenzymes. So far two publications have reported a TOP-poisoning effect of RSV in cells. Leone *et al.* [4] reported a strong poisoning potential of RSV in human glioma cells, while Baechler *et al.* [18] showed a weak poisoning potential in human epidermoid cells. To the best of our knowledge there are no data available regarding the TOP II poisoning potential of RSV in human colon cancer cells. However, data so far seem to indicate a cell type specific potential of the polyphenol on TOP II. 

A combination of 10 µM DOX with ≥100 µM RSV resulted in a significant decrease of the DOX-poisoning effects on TOP IIα. At ≥200 µM the cleavage complexes for TOP IIα were diminished by about 50% to a level not significantly different from the solvent control DMSO. The impact of RSV on the poisoning potential of DOX towards TOP IIβ was not as pronounced as for TOP IIα. A significant decrease of the level of DOX-stabilized cleavage complexes was only detected in the highest tested concentration of 250 µM RSV displaying a reduction of ~40%. This apparent isoenzyme-specific tendency might result from the known preference of DOX towards TOP IIα [19]. Nevertheless, the results of the ICE assay clearly demonstrate that RSV affects the poisoning potential of DOX in HT-29 cells.

The underlying mechanism of interaction between RSV and DOX with respect to the formation of cleavage complexes is unclear. DOX itself acts by inhibiting the religation of the DNA during the catalytic cycle of the TOP II thereby trapping the enzyme at its substrate. Until today it has not been elucidated how RSV poisons the enzyme. Both compounds could therefore compete for the same target or even for the same sites of the TOP. This is a well-known effect for compounds that act as so called “catalytic inhibitors” on TOP like, for example, merbarone. This class of inhibitors does not lead to an increase in the formation of cleavage complexes but do also effectively block the catalytic activity of the enzyme. A combination of a poison as DOX with a catalytic inhibitor abrogates the effects of the poison on TOP II as both compounds compete for the enzyme [20,21]. The anthocyanidin delphinidin (DEL) for example has been reported to act as a catalytic inhibitor, decreasing the amount of formed cleavage complexes after incubation with DOX in a comparable extend as RSV [22]. Catalytic inhibitors can for example act in the interface between enzyme and DNA or inhibit the binding of ATP during the catalytic cycle of TOP II and are discussed to be used in parallel during chemotherapy to prevent non-carcinogenic tissue from the damaging effects of TOP poisons [20]. As a parallel treatment with RSV and DOX leads to the decrease in cleavage complex formation combinatory incubation might also influence the potential of DOX to induce enzyme-mediated DNA damage. Poisoning of TOP II leads to severe consequences with respect to DNA-dependent processes. Transcription and replication are effectively blocked, while the risk of torsional stress is rising and the likelihood for DNA strand breaks increases [7]. The suppression of the TOP-poisoning potential of DOX raises the question on the consequences for DNA integrity and antineoplastic effectiveness of DOX. If RSV indeed suppresses the formation of cleavage complexes in cells a decrease in DNA damage might be expected.

### 2.2. Strand Breaking Potential

**Figure 2:**
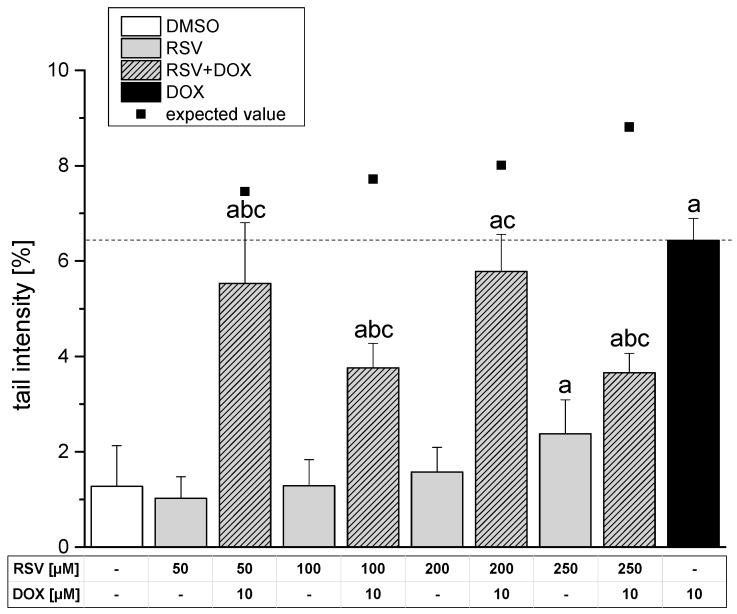
Impact of the single compounds and of combinatory treatments of RSV and DOX on DNA integrity in HT-29 cells in the comet assay. Cells were treated with the single compound RSV 1.5 h or pretreated with RSV in the indicated concentrations for 30 min and subsequently co-incubated together with 10 µM DOX or incubated with the single compound DOX for 60 min. DMSO (1% v/v) served as solvent control. DNA damage is expressed as amount of nuclear DNA found in the comet tail (“tail intensity”). Statistical analysis was done by One-way ANOVA followed by Fisher’s least significant difference (LSD) test. Significances indicated as “a” refer to a comparison to DMSO, significances indicated as “b” to a comparison to DOX, significances indicated as “c” to a comparison of a RSV treatment with or without DOX in the respective concentration. All significances refer to a statistical level of *p* < 0.05.

The impact of RSV on the DNA-damaging potential of DOX was monitored by single cell gel electrophoresis (comet assay) (Figure 2). After one hour of incubation with DOX tail intensity increased significantly to ~6% in comparison to the solvent control (Figure 2). RSV itself enhanced the level of DNA strand breaks slightly (~2% tail intensity) but significantly at a concentration of 250 µM. The combinatory treatment with ≥50 µM RSV diminished the level of DOX-mediated DNA strand breaks significantly with a maximum protective effect at 100 µM RSV (~4% tail intensity), but not in a strict concentration-dependent manner. At 200 µM RSV an apparent recurrence of DNA damage was observed followed by a repeated decrease of DNA damage by combination of 250 µM RSV and DOX. The comparison of the calculated expected values and the measured values for the co-incubation of RSV and DOX revealed significant differences being most pronounced for a combination of DOX with 100 µM (*p* = 0.0006) and 250 µM RSV (*p* = 0.0001) indicating antagonistic effects. With rising concentrations RSV appeared to counteract the DNA-strand breaking potential of DOX with an apparent maximum effect at 100 µM. This is in accordance with the results obtained in the ICE assay. The significant decrease in cleavage complex formation after a combinatory treatment of RSV and DOX might lead in the consequence to a suppression of TOP- mediated DNA damage. However, the lack of strict dose-dependency in the comet assay with an intermediate recurrence of DNA-damage by co-incubation with 200 µM RSV hints at an overlay of different effects. Probably, the intrinsic DNA-strand breaking potential of RSV at ≥200 µM comes into account, presumably compensating partly the protective effect. Such a u-shaped curve in the modulation of DOX-induced DNA damage by polyphenols was already published by Esselen *et al.* [22], who showed that DEL suppresses the DOX-induced cleavage complex formation of TOP II in HT-29 cells. As a consequence DEL counteracts the DNA-strand breaking potential of DOX up to a maximum protective effect. Higher concentrations of the anthocyanidin lead to an increase in tail intensity, which is assumed to be caused by the DNA damaging properties of the polyphenol itself. However, in the present study a different trend was observed with a combination of 250 µM RSV and DOX. Here, tail intensity was diminished again. A modulation in the cellular concentration of DOX might contribute to this phenomenon. If indeed RSV influences the uptake of DOX in that way that the DOX-uptake is decreased, the DNA-damaging effects might be preferentially affected by RSV causing only minor DNA damage after 1 h of incubation. A decrease in tail intensity in the comet assay as it is observed at the highest tested combination of 250 µM RSV and DOX might be a reasonable consequence.

### 2.3. Intracellular Concentration of DOX

**Figure 3:**
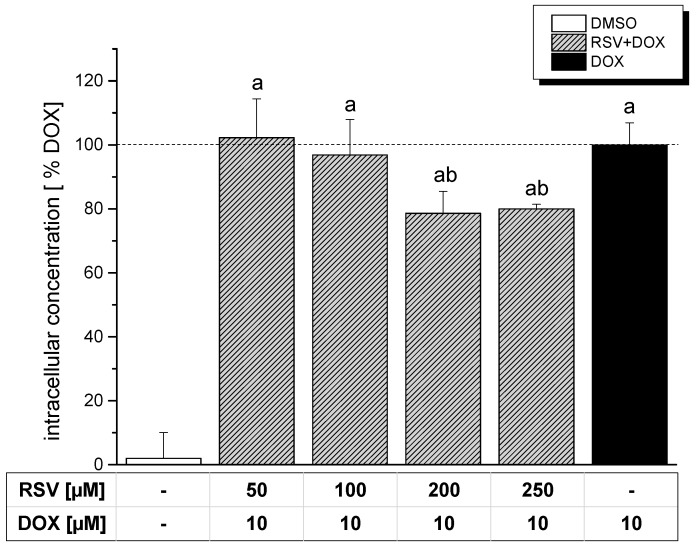
Intracellular concentration of DOX after 30 min of pre-treatment with RSV followed by 60 min of co-incubation in HT-29 cells. Intracellular concentration was assessed by fluorescence spectroscopy at a wavelength of λ_ex/em_ = 485/535 nm. Statistical analysis was done by One-way ANOVA followed by Fisher’s least significant difference (LSD) test. Significances indicated as “a” refer to a comparison to DMSO, significances indicated as “b” to a comparison to DOX. All significances refer to a statistical level of *p* < 0.05.

The intracellular concentration of DOX after co-incubation with RSV was assessed by fluorescence spectroscopy at a wavelength of λ_ex/em_ = 485/535 nm (Figure 3). RSV itself influenced the DOX signal at this wavelength in a negligible way (data not shown).

Co-incubation of DOX with ≥200 µM RSV was found to decrease the intracellular concentration of DOX (Figure 3), whereby the underlying mechanism (e.g., decreased uptake or enhanced efflux) has not been elucidated so far. Nevertheless, this decline of the intracellular concentration might be indeed of relevance for the effectiveness of DOX on cleavage complex stabilization as well as on DNA strand breaking potential. However, a decrease in DOX-mediated cleavage complex stabilization monitored in the ICE assay (Figure 1) was already observed at concentrations of 100 µM. The impact of RSV on the intracellular DOX concentration is therefore unlikely to be solely responsible for the suppression of the TOP II poisoning potential of DOX. Nevertheless, the decrease of the intracellular DOX concentration might indeed contribute to the decline of DOX-mediated DNA-damage in the presence of 250 µM RSV. The strand breaking effects observed at this concentration might indeed be preferentially influenced by RSV and might therefore explain the decline in tail intensity as the impact of DOX on DNA damage is diminished due to a decreased intracellular concentration.

So far, an opposite effect of RSV on the cellular concentration of DOX was reported in several studies. An increase in the cellular concentration of DOX was observed in human breast cancer cells [9,23,24], HepG2 and HELA cells [23]. In part, this was explained by a reduction of the permeability- glycoprotein (P-gp) pump activity and expression. However, HT-29 cells are known to express rather low levels of P-gp [25]. The influence of this parameter might therefore be negligible. A decreased cellular concentration in this cell type could be caused by cytotoxic effects influencing e.g., membrane integrity or cellular metabolism. We therefore investigated the impact of co-incubation on cytotoxicity.

### 2.4. Cytotoxicity

**Figure 4:**
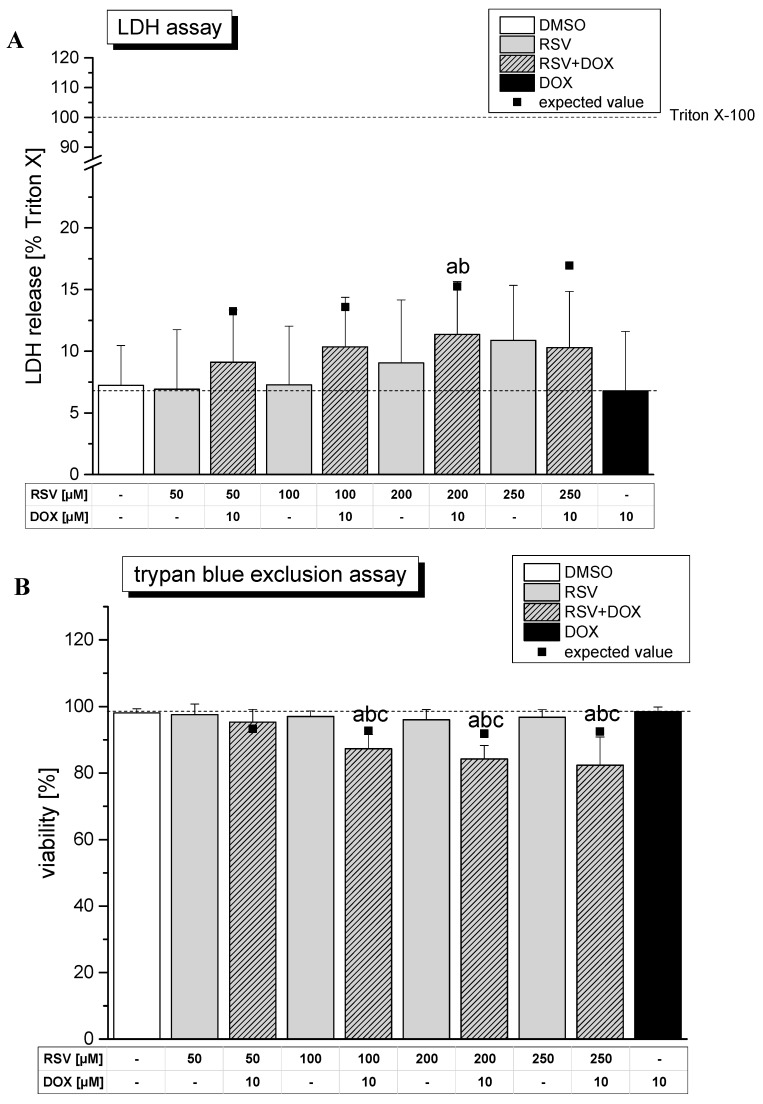

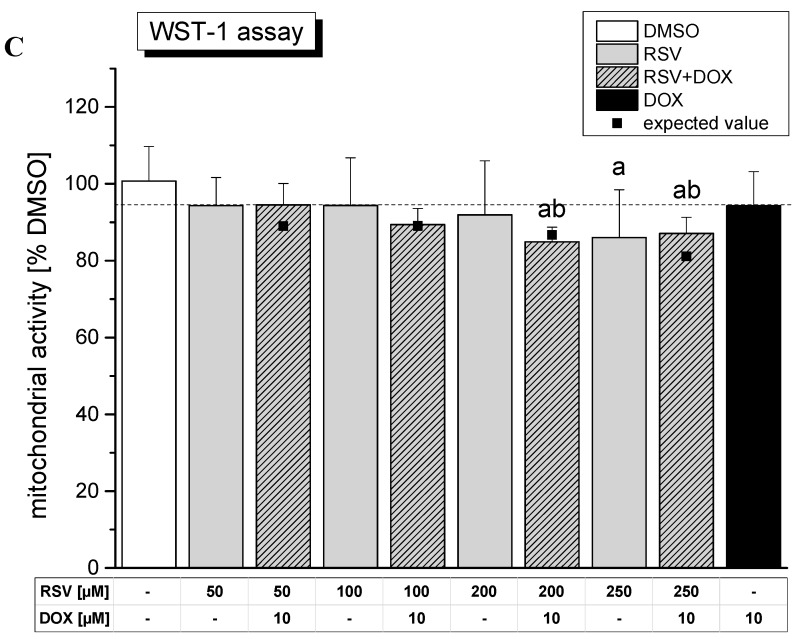
Cytotoxicity studies of incubation of RSV and DOX as single compounds or in combination (30 min pre-treatment with RSV or solvent, followed by 60 min co-incubation) in HT-29 cells. The lactate dehydrogenase (LDH) assay (**A**) as well as the trypan blue exclusion assay (**B**) monitor membrane integrity of the cells, while the WST-1 assay (**C**) was used to assess mitochondrial activity. The solvent DMSO (1% v/v) served as negative control. Statistical analysis was done by One-way ANOVA followed by Fisher’s least significant difference (LSD) test. Significances indicated as “a” refer to a comparison to DMSO, significances indicated as “b” to a comparison to DOX, significances indicated as “c” to a comparison of a RSV treatment with or without DOX in the respective concentration. All significances refer to a statistical level of *p* < 0.05.

In order to monitor potential cytotoxic effects of the single compounds and of the combinations three different screening assays were conducted: the lactate dehydrogenase assay (LDH), the trypan blue exclusion assay and the WST-1 assay (Figure 4).

The LDH assay as well as the trypan blue exclusion assay provide information on the integrity of the cell membrane. The former one measures the release of the cytosolic enzyme LDH in the supernatant while the latter one monitors the cellular exclusion of the dye trypan blue. Incubation of HT-29 cells with DOX alone for 60 min did not affect the LDH content of the supernatant. Only a combination of 200 µM RSV and 10 µM DOX significantly enhanced LDH activity by approx. 12%, but the observed increase differed not significantly from the effect of RSV as a single compound (Figure 4A). The trypan blue exclusion assay showed that treatment with DOX or RSV alone does not influence the viability of HT-29 cells under these conditions. In contrast to the results of the LDH assay, co-incubation of DOX with ≥100 µM RSV significantly decreased viability (Figure 4B). 

In the WST-1 the activity of mitochondrial dehydrogenases is assessed as a measure for cytotoxicity In accordance with the LDH assay and the trypan blue exclusion assay, treatment with DOX as a single compound for 60 min did not affect mitochondrial activity (Figure 4C). The single compound RSV significantly influenced mitochondrial activity of HT-29 cells only in the highest tested concentration of 250 µM. However, treatment with DOX in combination with different concentrations of RSV modulated the metabolic activity of the cells. Co-incubation with ≥200 µM RSV resulted in a significant decrease in mitochondrial activity (~85%) and differed significantly from treatment with DOX alone (~95%). Comparison of the results of the co-incubation experiments with calculated expected values based on the effects of the single compounds revealed a significant difference only for the combination of 250 µM RSV in the LDH assay (*p* = 0.036) and for 200 µM RSV (*p* = 0.040) in the trypan blue assay. As the significant differences between expected values and measured values are rather weak and do not show a continuous effect in the three different assays the results indicate an additive effects of the two compounds with respect to cytotoxicity after short-term incubation.

In summary, only very weak cytotoxic effects were detected by three different assays for the single compounds after short-term incubation even after incubation with RSV concentrations as high as 250 µM. This is in concordance with other publications showing that HT-29 cells are rather insensitive to potential cytotoxic effects of RSV [26,27]. Juan *et al.* [28] for example found no cytotoxic effects after 3 h of incubation with 400 µM RSV. In contrast to a treatment with the single compounds several cellular parameters of cytotoxicity were found to be influenced by the combination of RSV and DOX. The LDH assay and even more pronounced the trypan blue exclusion assay hint at a loss of membrane integrity/viability by treatment with a combination of RSV and DOX. Also the WST-1 assay indicated a decrease in mitochondrial activity after incubation of ≥200 µM RSV and DOX. In conclusion, the combination of DOX with RSV slightly enhanced the cytotoxic effects of the single compounds and indicates an additive effect in short-term toxicity for the combination of the polyphenol and the chemotherapeutic.

The impact on membrane integrity might be of relevance for the observed decrease of the intracellular concentration of DOX in combination with ≥200 µM RSV. Moreover, it cannot be excluded that these cytotoxic effects might influence the results obtained in the comet assay. However, the question arises if the modulation of cytotoxicity by RSV is also of relevance for the inhibition of cell growth by DOX.

### 2.5. Cell Growth Inhibition

**Figure 5:**
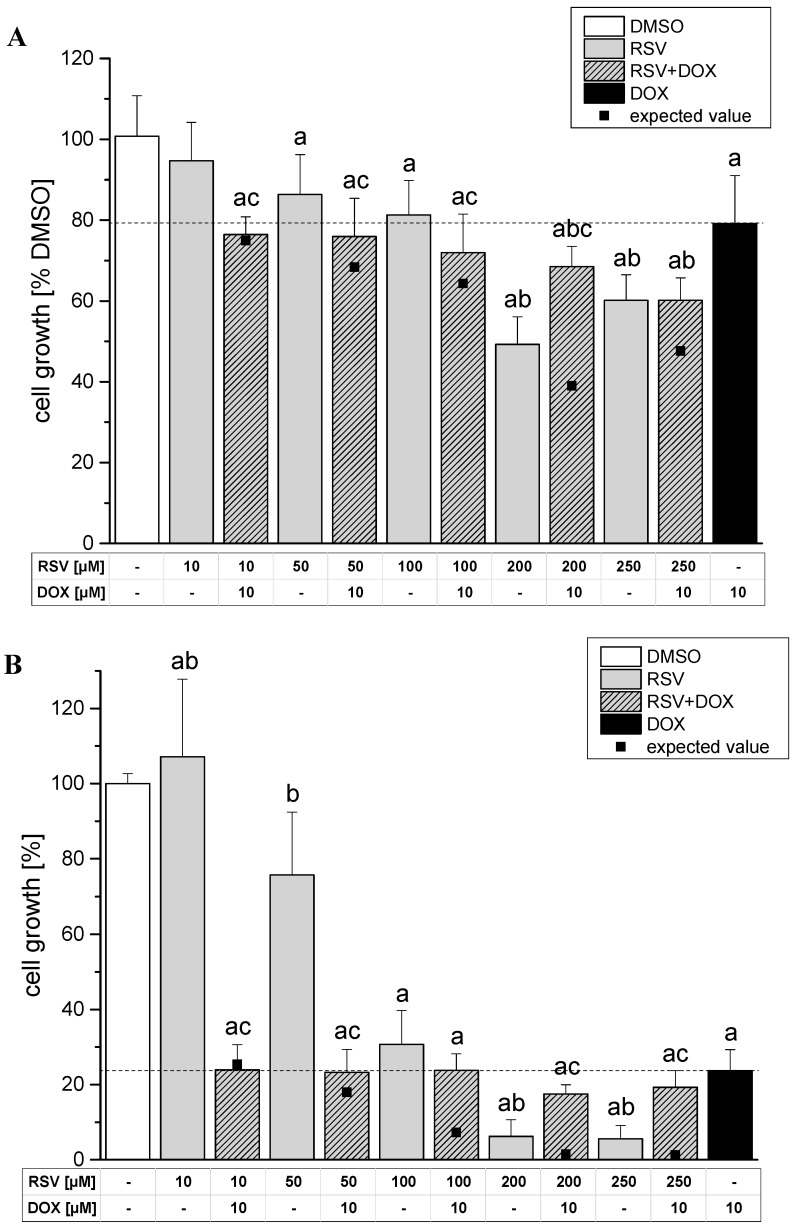
Growth inhibition monitored by sulforhodamine B (SRB) assay after incubation of RSV and DOX as single compounds or in combination in HT-29 cells. Cells were incubated with the single compounds or by a pre-treatment of RSV in the indicated concentrations for 60 min and then co-incubated together with 10 µM of DOX for additional (**A**) 23 h or (**B**) 71 h. The solvent DMSO (1% v/v) served as negative control. Statistical analysis was done by One-way ANOVA followed by Fisher’s least significant difference (LSD) test. Significances indicated as “a” refer to a comparison to DMSO, significances indicated as “b” to a comparison to DOX, significances indicated as “c” to a comparison of a RSV treatment with or without DOX in the respective concentration. All significances refer to a statistical level of *p* < 0.05.

The impact of RSV on the growth inhibitory effects of DOX was studied in the sulforhodamine B (SRB) assay. The cells were pre-incubated with RSV for 60 min, followed by 23 h or 71 h of co-incubation with DOX (Figure 5).

After 24 h incubation with RSV cell growth was significantly reduced in concentrations ≥50 µM (Figure 5A). The results of the co-incubation differ significantly from the treatment with the single compound RSV with one exception at the highest concentration of 250 µM. However, the extent of growth inhibition achieved by co-incubation was significantly lower than the expected value of additive effects of the single compounds at 200 µM (*p* = 0.0008) and 250 µM (*p* = 0.046) RSV + DOX , which indicates an abrogation of the additive effect observed in concentrations ≤100 µM.

After 72 h incubation with RSV resulted in a potent concentration-dependent decline of cell growth up to a maximum growth inhibition of ~5% at 250 µM, which is comparable to previous results obtained in HT-29 cells [27,28] whereas after co-incubation the growth inhibition appeared to remain static at a level of about 20% and seem to reflect preferentially the growth inhibitory potential of DOX (Figure 5B). At higher RSV concentrations (≥100 µM RSV) the cell growth inhibition in combination with DOX was significantly lower than the expected value based on the sum of the effects of the single compounds (*p* = 0.0164 for 100 µM; *p* = 0.0036 for 200 µM; *p* = 0.0014 for 250 µM). Of note, in that concentration range RSV had a significant stronger impact on cell growth than the combination of the polyphenol and the chemotherapeutic.

By trend the results of 24 h of incubation were comparable to the results of the short-term cytotoxicity experiments. The combination appeared to be more toxic in the presence of RSV than the single compounds. However, this trend was not further accomplished by the data obtained after 72 h of incubation. Here, the presence of DOX appeared to diminish the growth inhibitory potential of RSV. Comparison of the expected values calculated as the sum of the effects of the single compounds showed that a combination of high concentrations of RSV (≥200 µM) and DOX abrogates the additive effects observed at lower concentrations. This is in contrast to reports on combinatory effects of RSV and DOX in human breast cancer cells, where a synergistic mode of action for RSV and DOX is proposed [9,24]. On the other hand, Al-Abd *et al.* [23] reported a synergistic interaction of RSV and DOX on the cell growth of human breast cancer cells, but additive effects on human cervical adenocarcinoma and hepatocarcinoma cells after 72 h of incubation. Further on, studies focusing on cardiomyocytes revealed a protective effect of RSV on DOX-induced toxicity [14]. To our knowledge, no data on the effects of respective combinations in colon cells is available so far. The results published on other cell types hint at a cell-type dependent mode of interaction, which might explain the diverging results obtained in HT-29 cells where short-term effects implies an additive effect, while the impact of RSV on cell growth inhibition seems to be negligible.

The present data demonstrate that in HT-29 colon carcinoma cells RSV antagonizes the TOP-poisoning effectiveness of DOX as well as the DNA-damaging potential. The decrease of the DOX poisoning potential by RSV might be caused by RSV acting rather as catalytic inhibitor than as poison in HT-29 cells. The chemotherapeutic aclarubicin for example is also known to be capable of poisoning TOP I and of inhibiting the enzyme catalytically, while the way of action is dependent on the concentration applied. Catalytic inhibitors are clinically of relevance as they are reported to reduce severe side effects of TOP poisons during chemotherapy [20]. The data of this work indicates a potential preventive effect of RSV on colonic tissue with respect to DNA damaging properties and TOP poising of DOX. Unfortunately, this effect is not further accomplished by the data according to cytotoxic effects. Despite of the reduced TOP poising potential of DOX a combinatory treatment of DOX and RSV indicates an additive cytotoxic effect after short-term exposure and only negligible influence of RSV on cell growth. Further molecular pathways might therefore be involved. DOX is for example also known to generate ROS [29,30], while RSV as many polyphenols shows an antioxidant potential in lower concentrations and a prooxidative potential in higher concentrations [31]. It has been reported that the antioxidant potential of RSV might counteract the cardiotoxic potential of DOX [12,13,14]. Interestingly, previous work reported an induction of apoptosis by RSV through the generation of ROS in mitochondria in HT-29 cells [28,32]. Further on, very recent work revealed an involvement of TOP IIβ in DOX- mediated cardiotoxicity [33]. 

Co-exposition of RSV with DOX might arise from the development of combinatory strategies of promising bioactive natural compounds with classical chemotherapeutics. However, considering the increasing popularity of RSV as already available food supplement, intake of respective supplements by patients under chemotherapy might occur. In the present study, interference of RSV with DOX effectiveness was observed in a concentration range exceeding 50 µM RSV, thus exceeding by far the expected plasma concentration, which does not exceed ~5 µM even after oral doses as high as 5 g [34,35]. However, despite known low systemic bioavailability of RSV, locally in the gastrointestinal tract bioactive concentrations might occur after supplement consumption. Patel *et al.* [36] reported a significant reduction of tumor cell proliferation in colon cancer patients after oral intake of RSV. In addition, recently oral intake of blackberry extract was demonstrated to suppress the TOP-poisoning and DNA-damaging potential of i.p. applied irinotecan in Wistar rats [37], demonstrating that orally applied bioactive natural products with low systemic bioavailability might provide protective properties against the genotoxic potential of systemically applied chemotherapeutics in colonic tissue. 

## 3. Experimental Section

### 3.1. Chemicals

All chemicals were purchased from Carl Roth GmbH + CO. KG (Karlsruhe, Germany) and from Sigma-Aldrich Chemie GmbH (Taufkirchen, Germany) or as indicated separately. 

### 3.2. Cell Culture and Treatment

The human colon cancer cell line HT-29 was purchased from the Leibnitz Institute DSMZ- German Collection of Microorganisms and Cell Cultures (Braunschweig, Germany). The cells were kept at 37 °C and 5% CO_2_ under humidified atmosphere. The culture of the cells was carried out in DMEM supplemented with 10% (v/v) heat-inactivated fetal calf serum (FCS) and 1% (v/v) penicillin/streptomycin (Invitrogen^TM^ Life Technologies, Karlsruhe, Germany). Treatment of the cells was done in serum-free medium for the short-term incubations and with serum-supplemented medium for the long-term incubation experiments, with a final solvent control concentration of 1% (v/v) DMSO. For co-incubation experiments the cells were pre-treated with different concentrations of RSV for 30 min and thereafter co-incubated for 60 min with 10 µM of DOX and RSV. Long-term incubation was done by 60 min of pre-treatment followed by 23 h or 71 h of co-incubation. The concentration range for RSV was chosen based on preliminary studies.

### 3.3. Isolating in Vivo Complexes of Enzyme to DNA Assay (ICE Assay)

For the monitoring of the TOP inhibiting potential the ICE assay was performed as described by Subramanian *et al.* [38] with slight modifications. Here, 5 × 10^6^ cells were seeded in 15 cm Petri dishes and allowed to attach for 48 h. After treatment the cells were harvested by the addition of lysis buffer (10 mM Tris, pH 8.0, 1 mM EDTA, 1% w/v N-lauroylsarcosin). The cell lysate was laid on the cesium chloride gradient and centrifuged for 24 h at 100,000× *g* in order to separate free TOP II from TOP II bound to the DNA (cleavage complexes). Thereafter, each sample was split into 20 fractions and the DNA content was measured by a NanoDrop 2000c Spectrophotometer (Thermo Fisher Scientific, Waltham, MA, USA). The TOP II complexes are found in the same fractions as the DNA (usually between fraction 8-11). These DNA-rich fractions were blotted on a nitrocellulose membrane and the complex formed by TOP II and DNA were detected by chemiluminescence measurements using antibodies against TOP IIα (H-231) and TOP IIβ (H-286) as first antibodies and a compatible HRP-conjugated antibody as second antibody (Santa Cruz Biotechnology, Dallas, TX, USA). The quantification of the arbitrary light units signal was done with the software Multi Gauge V 3.2 (Fujifilm, Tokyo, Japan) by referring to the control DOX.

### 3.4. Comet Assay

To detect potential DNA damaging or protective effects after co-incubation of RSV and DOX the single strand gel electrophoresis assay (comet assay) was performed as described by Tice *et al.* [39] with slight modifications. Shortly, 3 × 10^5^ cells were seeded in Petri dishes (diameter of 5 cm) and allowed to grow 48 h. After incubation the cells were harvested by trypsin treatment and viability as well as cell number was assessed using the trypan blue exclusion assay. An aliquot of 3 × 10^4^ cells per sample were embedded in 0.8% (w/v) low melting agarose and transferred on a slide. Cell lysis was performed in a buffer containing 89 mL lysis buffer stock solution (2.5 M NaCl, 100 mM EDTA, 100 mM Tris pH, 1% w/v N-lauroylsarcosin, pH 8.0), 10 mL DMSO and 1% (v/v) Triton-X 100 for 24 h at 4 °C. Thereafter the samples were allowed to equilibrate in the electrophoresis buffer (200 mM NaOH, 1 mM EDTA, pH 13.5) for 20 min before starting the electrophoresis (20 min, 300 mA). After staining of the cells with 10 µg/mL ethidium bromide solution the tail intensity (amount of nuclear DNA found in the tail) was determined by the software CometIV (Perceptive Instruments, Suffolk, UK). 

### 3.5. Intracellular Concentration of DOX

For assessing the potential modulation of the intracellular concentration of DOX a plate reader based method according to Wong *et al.* [40] was performed. Here, 3 × 10^4^ cells were seeded in each well of a 96-well plate and allowed to attach for 48 h. After incubation the cells were washed twice with PBS and thereafter lysed by the addition of 100 µL of 1% (v/v) Triton-X 100 in PBS. After 10 min of shaking the fluorescent signal was measured at a wavelength of λ_ex/em_ = 485/535 nm. For quantification a standard curve of DOX was prepared by mixing the cell lysate of untreated cells with different concentrations of DOX. The background fluorescence was assessed by measuring the fluorescence signals of wells that contained untreated cells. In addition, a potential quenching of the DOX signal by RSV was monitored by measuring the fluorescence signal of a mixture of different RSV concentrations and DOX dissolved in PBS. The calculation of the intracellular concentration was done by referring to the control DOX. The data is therefore given as percentage of DOX treated cells.

### 3.6. Cytotoxicity

In order to evaluate the impact of RSV on the toxicity of DOX after 1.5 h of incubation three different cytotoxicity assays were performed. 

#### 3.6.1. Lactate Dehydrogenase Assay (LDH- Assay)

For the LDH assay 3 × 10^4^ cells were seeded in each well of a 96-well plate and allowed to attach for 48 h. After incubation the supernatant was transferred to a new 96-well plate and the amount of enzyme in the supernatant was assessed by the commercial available LDH kit (Roche Applied Science, Mannheim, Germany). After photometrical measurements at a wavelength of λ = 490 nm analysis was done by calculating the percentage of LDH activity in the supernatant in relation to the positive control Triton X-100 (1% v/v), which was set to 100%.

#### 3.6.2. Trypan Blue Exclusion Assay

For the trypan blue exclusion assay 3 × 10^5^ cells were seeded in Petri dishes and allowed to grow for 48 h. After incubation cells were harvested by trypsin treatment and incubated in trypan blue solution for 2 min in a proportion of 1:10. The viability was assessed by manual counting the overall number of cells and the ones that were stained blue (interpreted as “dead”).

#### 3.6.3. WST-1 Assay

The mitochondrial activity of the cells was monitored with the help of the commercial available WST-1 test kit (Roche Applied Science). As described in the protocol of the kit an appropriate number of cells (3 × 10^4^ per well) were seeded in a 96-well plate. After incubation the cells were washed twice with PBS and incubated for 20 min at 37 °C with 10 µL of the WST-1 reagent solved in 100 µL of serum-free DMEM. Thereafter the mitochondrial activity of the cells was assessed by photometric measurements at a wavelength of λ = 490 nm using a plate reader (Victor^3^ V, PerkinElmer, Waltham, MA, USA). Analysis was done by calculating the percentage of mitochondrial activity in relation to the solvent control DMSO. Triton-X 100 (1% v/v) served as positive control.

### 3.7. Cell Growth Inhibition (Sulforhodamine B Assay)

To investigate long term effects of a combination of RSV and DOX on the proliferation of HT-29 cells the sulforhodamine B (SRB) assay was conducted. As HT-29 cells have a doubling time between 40 to 60 h according to the Leibnitz Institute DSMZ (German Collection of Microorganisms and Cell Cultures) the assay was performed after 24 h and 72 h of incubation. For the 24 h incubation duration 1 × 10^4^ cells were spread into each well of a 96-well plate. For 72 h of incubation 3 × 10^3^ cells were used. Cells were allowed to attach for 48 h. Thereafter the cells were incubated with the respective concentration of RSV. For the co-incubation experiments cells were treated with RSV for 60 min, followed by a co-incubation with RSV and DOX or by a single treatment with DOX for 23 h or 71 h. Incubation was carried out in medium supplemented with 10% fetal bovine serum to assure proper proliferation conditions. After cell fixation by the addition of 10 µL of trichloroacetic acid (50% w/v) the cell protein was stained by the addition of 0.4% (w/v) sulforhodamine B solution for 1 h. After drying the dye was dissolved in Tris buffer (10 mM, pH 10.0) and a photometrical measurement was performed at a wavelength of 570 nm. The growth inhibitory effect was calculated as percentage of DMSO treated cells.

### 3.8. Statistics

Results are given as mean ± SD of at least four independently performed experiments. Statistical analysis was done by using the software OriginPro 9.0G (OriginLab Corporation, MA, USA). For normality testing the Shapiro-Wilk test was used (*p* > 0.05). A comparison of different groups was done by One-way ANOVA followed by Fisher’s least significant difference (LSD) test. A significance level of *p* < 0.05 was considered as significant and is illustrated in every figure.by letters.

The “expected value” was calculated after the concept of “response addition” by the addition of the measured values of the single compounds [41,42]: $expected\;value\;\left[\%\right]\;=\;mean_{subtance\;1}\;\left[\%\right]\;+\;mean_{subtance\;2}\;\left[\%\right]\;-\;mean_{subtance\;1}\;\left[\%\right]\;\times \;mean_{subtance\;2}\;[\%]$

The standard error of the mean (SEM) was calculated as follows: $SEM\;\;=\;\sqrt{{\left(SEM_{substance\;1}\right)}^{2}\;+\;{\left(SEM_{substance\;2}\right)}^{2}}$

The statistical comparison of the expected value with the measured value was done by performing the unpaired Student’s *t*-test. A statistical significant difference expresses either an effect that is less than the expected additive effect (measured value < expected value) or an effect that is more than the expected additive effect (measured value > expected value). No statistical significance is regarded as additive effect.

## 4. Conclusions

In this study we investigated the impact of RSV on the genotoxic and cytotoxic potential of DOX with special emphasis on TOP II. We are the first to show that RSV modulates the DOX-induced formation of TOP II-DNA complexes in cells. As a consequence, RSV also shows protective effects on DOX-induced DNA strand breaks, though the protective effect were found in a rather narrow concentration range. Further on, the polyphenol influences the intracellular concentration of DOX. This might be caused by the reported enhanced cytotoxicity after 1.5 h and 24 h of a combined incubation. However, the present study demonstrates that further investigations are needed to clarify whether consumption of RSV supplements during chemotherapy affects the effectiveness of the treatment and, if not so, whether RSV might be useful as a protective factor for the colonic tissue during therapy.
